# Hospital pharmacists’ mental health during home isolation in the post-pandemic era of COVID-19: influencing factors, coping strategies, and the mediating effect of resilience

**DOI:** 10.3389/fpubh.2024.1268638

**Published:** 2024-01-31

**Authors:** Zhao Yin, XiangYu Wang, Xiaojing Lu, Hang Fu

**Affiliations:** ^1^Institute for Hospital Management of Henan Province, Zhengzhou, China; ^2^Department of Pharmacy, The First Affiliated Hospital of Zhengzhou University, Zhengzhou, China; ^3^Beijing Chaoyang Hospital, Party Committee Office, Beijing, China; ^4^Henan Drug Clinical Comprehensive Evaluation Center, Zhengzhou, China

**Keywords:** home quarantine hospital pharmacists (HQHPs), COVID-19, embedded mixed methods, resilience, mediating effect, mental health

## Abstract

**Objective:**

There is a notable research gap on the mental health challenges faced by hospital pharmacists in the post-pandemic era. Therefore, the present study aims to explore mental health status, influencing factors, coping strategies, and the mediating effect of resilience of home quarantine hospital pharmacists (HQHPs).

**Methods:**

An embedded mixed-method study was conducted in Zhengzhou, a megalopolis in central China, from 20 October to 3 November 2022. For the qualitative study, semi-structured interviews and focus group discussions were conducted among HQHPs to understand their mental health state，the factors that affect their mental health, and the alleviating strategies they used. For the quantitative study, the Generalized Anxiety Disorder Scale (GAD-7) and the Chinese version of the Connor-Davidson Resilience Scale (CD-RISC-25) were used to explore the mental health level of HQHPs. Meanwhile, the mediating effect of resilience was explored and verified with the bootstrap method or the structural equation model.

**Results:**

20 HQHPs participated in the qualitative study. Two themes were identified in terms of the factors that influenced the HQHPs’ mental health levels. The positive factors include optimism, family company, and connection with friends, while the negative factors include inadequate supplies, inadequate information, and other emergencies. 210 HQHPs participated in the quantitative study, with a median resilience score of 66 [55.75, 74] in the lowest score range and an anxiety score of 5 [2, 7] in the mild anxiety range. The correlation analysis showed that nine of the 10 influencing factors identified in qualitative research were positively correlated with CD-RISC-25 scores and negatively correlated with the GAD-7 score. The mediating effect of resilience on anxiety was significant (95% bootstrap CI [−1.818, −0.384], *p*<0.001; or 95% bootstrap CI [−1.174, −0.430], *p*<0.001).

**Conclusion:**

Even in the post-epidemic era, hospital pharmacists face psychological challenges. They actively address these issues based on accumulated experience and a stabilized mindset. In this process, resilience plays a significant mediating role.

## Introduction

Coronavirus Disease 2019 (COVID-19) has left an indelible mark on community life, healthcare, and education (1, 2). Governments worldwide have responded with myriad measures, ranging from city lockdowns to social isolation, quarantines for infected populations, and home isolation (3). In particular, individuals working in healthcare settings, particularly hospital pharmacists and other healthcare workers (HCWs), are disproportionately susceptible to mental health challenges during this unprecedented crisis. China, being the first nation to face the challenges posed by COVID-19, implemented stringent quarantine measures to curb the spread of the virus within its borders and in other regions (4). After years of battling the COVID-19 virus and its variants, China’s domestic epidemic prevention and control program transitioned to a normalized stage in 2022. Despite this progress, local and sporadic virus outbreaks have recently emerged in specific regions and cities (5). An illustrative case is the recent COVID-19 outbreak in Zhengzhou, the capital of central China’s Henan Province, disrupted work for all practitioners. These individuals were compelled to stay home due to temporary lockdowns and controlled zones designed to stem the virus’s spread. This situation stands out as uncommon on a global scale, where many regions have eased their epidemic containment measures. However, despite the worldwide impact of such extreme conditions, research on the specific consequences for the mental health of pharmacists remains relatively limited.

Quarantine has been shown to significantly reduce mental health levels (6–8). Brooks et al. (9) reviewed the psychological impact of quarantine and isolation and found negative psychological effects, including post-traumatic stress symptoms, confusion, insomnia, avoidance behaviors and confusion, highlighting the importance of reducing quarantine to a minimally required period of time. In the battle against COVID-19, pharmacists have become a crucial component of the healthcare system. Their contributions include formulating professional service guidelines for pharmacists and pharmacies, creating emergency drug lists, addressing and monitoring drug shortages, offering telemedicine services, conducting public education on infection prevention and disease management, as well as actively participating in clinical trials and drug evaluations (10, 11). However, compared to nursing staff and physicians, the psychological challenges associated with COVID-19 for hospital pharmacists have received relatively less attention (12). Studies performed prior to the COVID-19 outbreak have shown that pharmacists have a lower health-related quality of life and higher levels of anxiety than the general population and other health professionals, including physicians, nurses, and others (13, 14).

Anxiety is a prominent marker in researching the psychological well-being of HCWs during the COVID-19 pandemic for several key reasons. First, studies report that more than 25% of HCWs, particularly medical personnel, experience anxiety at significant levels (15). Second, anxiety reflects an emotional response that includes concerns, tension, and fear regarding potential threats, which makes it crucial to capture the emotional state of HCWs navigating stress and uncertainty (16). Third, the levels of anxiety in HCWs can affect their focus on patient safety and job performance, contributing to a better understanding of their professional responsibilities during epidemic responses (17). Last, anxiety is commonly associated with stressful events. Given the global impact of the COVID-19 pandemic, it serves as a critical marker for characterizing mental health levels in studying the psychological issues of healthcare professionals post-quarantine in the epidemic’s aftermath.

Dealing with special situations arising from the epidemic, such as quarantine, is an essential concern in the post-epidemic era. Previous studies have reported a variety of coping strategies, such as establishing harmonious interpersonal relationships, building a good community environment, and creating a good climate of public opinion (18). These external pillars have played an instrumental role in surmounting adversities amidst the pandemic (19). In addition, personal factors such as a positive mindset, optimism, and inner drive are also important, with resilience being one of the individual inner drives that have been particularly highlighted in previous reports (16). Resilience, a critical concept in positive psychology, is the personal ability to cope with or overcome various perceived adversities and stress. Individuals with higher levels of resilience work through challenges using personal resources, strengths, and other positive psychological capital capacities, such as hope, optimism, and self-efficacy, to “bounce back” as a stronger person than before (20). Previous research has shown that physicians’ and nurses’ stress mindset and resilience could mitigate the negative outcomes of COVID-19 health anxiety by helping them view this stressful situation more positively and consider it a challenge rather than a threat (21). Hence, we seek to employ psychological resilience as a mediating variable to explain the possible intrinsic regulatory determinants that affect the response of HQHPs to anxiety.

Despite extensive research on healthcare workers’ coping strategies, a significant research gap exists in understanding how HQHPs cope, particularly in the post-epidemic era. Examining the entire COVID-19 timeline, healthcare workers face distinct challenges requiring nuanced coping strategies from the pandemic through the transition period to the post-epidemic era. During the pandemic, prioritizing teamwork, providing practical support tools, and ensuring comprehensive pharmacist training are crucial to managing daily high-intensity pressure and fatigue (22–24). In the transition period, in addition to adaptation training and mental health support, open communication and self-management are vital to adapting to a new workplace and promoting mental well-being (25). In the post-epidemic era, healthcare workers may experience fatigue and emotional stress, requiring mental health support services, such as professional consultations and participation in support groups, along with promoting adequate rest and recovery time (26). For HQHPs in mainland China during the post-epidemic era, despite the lenient global epidemic prevention policies, they are obligated to undergo home isolation, leading to increased mental stress, physical stress, and increased work burden (26). Consequently, the mental health challenges faced by hospital pharmacists in mainland China during home isolation require urgent attention and investigation (27).

### Study aims

Given the unique circumstances outlined above, there is a critical need to understand the mental health status of HQHPs, identify influencing factors, explore coping strategies, and uncover potential intrinsic regulatory factors. In response to these considerations, this study focuses on anxiety as the outcome variable linked to mental health levels, with resilience serving as a mediator. The research aims to accomplish the following objectives: (1) clarify the current psychological well-being of HQHPs during home quarantine in the post-epidemic era; (2) analyze potential factors affecting the mental health levels of HQHPs, emphasizing the mediating role of psychological resilience; and (3) investigate potential strategies employed by HQHPs in addressing mental health issues.

## Methods

### Study design

Previous research in this field mainly relied on cross-sectional quantitative studies or qualitative investigations through in-depth interviews. While each method has strengths and limitations, our study employs a mixed-method embedded research design to explore mental health among HQHPs (28). This design seamlessly integrates both qualitative and quantitative methodologies, allowing for a comprehensive examination of the research question. By synthesizing data from these two approaches, we aim to capture information on multiple levels and dimensions, enriching our understanding of the research phenomenon. An advantageous aspect of this approach is the mutual validation between qualitative and quantitative components (29).

**Figure 1:**
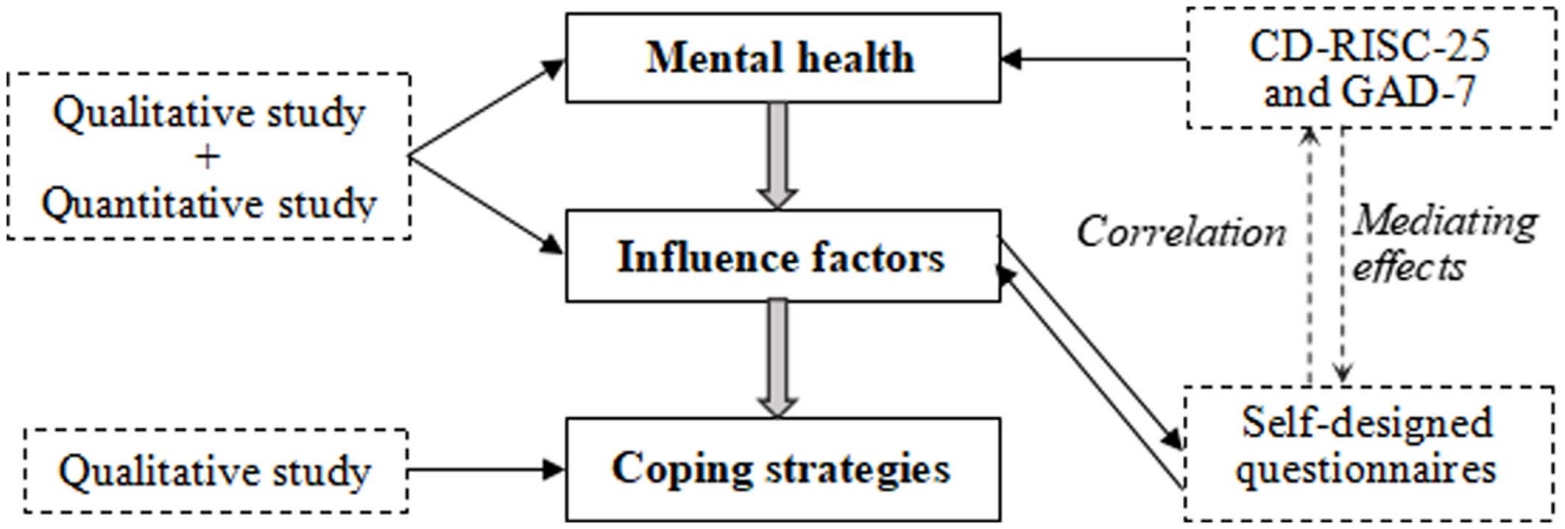
Research methodology.

To achieve our research objectives, we followed the procedural steps outlined in mixed-method embedded research (30). First, we used qualitative research to gain an in-depth understanding of the psychological experience of quarantined hospital pharmacists and the multidimensional factors influencing their mental health in this exceptional situation, as well as to summarize their coping strategies. Second, a quantitative study was performed to validate the factors identified during the qualitative study and analyzed the mediating role of resilience. Finally, the results of the qualitative and quantitative studies were integrated. The detailed flow chart of the study is presented in Figure 1.

### Settings and participants

HQHPs (20 October to 30 November 2022) were selected from several large hospitals in Henan province. These sample hospitals included the First Affiliated Hospital of Zhengzhou University, the Second Affiliated Hospital of Zhengzhou University, the Third Affiliated Hospital of Zhengzhou University, the 7th People’s Hospital of Zhengzhou, Henan Children’s Hospital, Henan Provincial Cancer Hospital, and Henan Coking Coal Central Hospital. With a combined capacity of 20,700 beds and approximately 730 pharmacists, these traditional large tertiary hospitals ranked among the best in central China (31). The inclusion criteria for the participants were (1) volunteered to participate in this study, (2) worked a minimum of 1 year in the department of pharmacy in the sample hospitals, and (3) had experienced quarantine between 20 October and 3 November 2022.

This study used a census approach, sampling all eligible HQHPs from the sample hospitals. The researcher communicated with the pharmacy department leaders in the sample hospitals to discuss the research objectives and plans. Subsequently, all eligible HQHPs were recruited by the pharmacy department leaders in their respective institutions. Finally, 240 pharmacists participated in this study, including clinical pharmacists, dispensing pharmacists, managers, information pharmacists, etc. A total of 240 questionnaires were sent, and 210 valid questionnaires were recovered (87.50%). Of the 210 participants in the quantitative study, 20 volunteered to participate in a qualitative interview study. Eight pharmacists participated in the one-on-one interview and 12 participated in focus group discussions (four in each group). Participant flow is shown in Supplementary Figure S1, and the demographic characteristics of all the participants are shown in Table 1.

**Table 1:** Demographic characteristics of all participants.

Item	Classification	Qualitative study	Quantitative study
		*Number (%;n = 20)*	*Number (%;n = 210)*
Gender	Male	5 (25.00)	52 (24.76)
	Female	15 (75.00)	158 (75.24)
Age	≤35	10 (50.00)	122 (58.10)
	36–50	6 (30.00)	71 (33.80)
	>50	4 (20.00)	17 (8.10)
Educational level	Technical secondary school	0 (0.00)	17(8.10)
	Junior college	1 (5.00)	19(9.05)
	Undergraduate	9 (45.00)	106 (50.47)
	Postgraduate	10 (50.00)	68 (32.38)
Working years	<10	9 (45.00)	107 (50.95)
	≥10	11 (55.00)	103 (49.05)
Marriage status	Unmarried	1 (5.00)	40 (19.05)
	Married	18 (5.00)	165 (78.57)
	Divorced	1 (5.00)	5 (2.38)
Professional title	Junior professional	0	54 (25.71)
	Intermediate professional	13 (65.00)	127 (60.48)
	Associate senior professional	6 (30.00)	23 (10.95)
	Senior professional	1 (5.00)	6 (2.86)
Days of continuous quarantine	0–7 days	5 (25.00)	81 (38.57)
	8–15 days	15 (75.00)	129 (61.43)

### Instruments

#### Resilience

The Chinese version of the Connor-Davidson Resilience Scale (CD-RISC-25) (20, 32), was used to evaluate individual resilience. This valid and reliable scale (Cronbach’s alpha = 0.963) comprises 25 items rated on a five-point Likert scale from 0 (not true at all) to 4 (true all the time). The total score ranges from 0 to 100, with a higher score indicating greater resilience. The scores for the lowest quartile range from 0 to 73, the second lowest quartile scores range from 74 to 82, the third quartile scores range from 83 to 90, and the fourth quartile scores range from 91 to 100 (33).

#### Anxiety

Anxiety levels were assessed using the 7-item Generalized Anxiety Disorder Scale (GAD-7), a well-validated tool with high reliability (Cronbach’s alpha = 0.929). This instrument aggregates responses from seven questions designed to screen and diagnose generalized anxiety disorder (34). The respondents provide 4-point responses, ranging from 0 (not at all) to 3 (nearly every day), both in clinical practice and research. The cumulative scores, ranging from 0 to 21, are categorized as follows: normal (0–4), mild (5–9), moderate (10–14), and severe (15–21) anxiety. Participants with a total GAD-7 score ≥ 10 are identified as positive for anxiety screening (35).

#### Self-designed questionnaire

In the absence of available standardized questionnaires, a self-designed questionnaire was developed comprising 10 questions, guided by the influencing factors identified in the preliminary findings of the qualitative study (Supplementary Table S1). The self-designed questionnaire demonstrated satisfactory internal consistency, with Cronbach’s alpha at 0.839. To ensure the clarity and completeness of the questions, a pilot study was conducted involving 20 HQHPs. The questionnaire was evaluated based on feedback received. After incorporating the suggestions provided by the participating HQHPs and making the necessary adjustments, the primary research was initiated.

### Data collection and analysis

#### Qualitative study

For the qualitative survey, purposeful sampling was used to obtain participants from the HQHPs involved in the quantitative research. Due to the restrictions of the COVID-19 pandemic, all interviews were conducted by phone calls or video chat. After informed consent, semi-structured interviews or focus group discussions were led by one researcher (ZY) and recorded by another (XJL or XYW). An interview topic guide (Supplementary Table S2, designed by ZY and XJL before the interview) was piloted in an interview. Data saturation was reached after interviewing 8 HQHPs and three focus group discussions. All interviews took place from 20 October to 3 November 2022. Data saturation was defined as “no new themes or codes emerge from interviews.”

The interview data were analyzed using a thematic analysis approach, a method to identify, analyze, and report patterns within the data widely used in qualitative research. The key steps include familiarization with the data, generating initial codes, searching and reviewing the themes, naming the themes, and producing the final report (36). Within 24 h after the interview, the interview data were transcribed verbatim by one researcher (ZY) and checked by two other researchers (XJL and XYW). After confirming that the transcriptions were correct, two researchers (ZY and XJL) independently completed the extraction of the codes and wrote the first draft of the themes and subthemes. Subsequently, the research group discussed the first draft and proposed comments and suggestions for theme structure and language expression. Under the guidance of a senior research expert, the research team compared and analyzed discrepancies and established the final themes and subthemes. The final draft were developed with the agreement of the research group, and appropriate representative quotes were selected to present the themes or subthemes. NVivo12 software was used to manage and analyze the data in the process described above. This study followed the Consolidated Criteria for Reporting Qualitative Research (COREQ) checklist (Supplementary Table S3) (37).

#### Quantitative study

For the quantitative study, the responses to the online survey were exported from Questionnaire Star to an Excel file (Excel version 2016; Microsoft Corp., Redmond, WA, United States). Data were cleaned, coded, and analyzed using IBM SPSS® (Armonk, NY, United States) statistical software version 26. Measurement data are expressed as median with [25% lower quartile; 75% upper quartile], and count data are expressed as percentages. Each participant’s total score for CD-RISC-25 and GAD-7 was calculated by summing the individual item scores. The Shapiro–Wilk test was used to assess data normality. Independent sample t-test or one-way ANOVA was used to examine differences in CD-RISC-25 and GAD-7 among demographic variables following a normal distribution. The Mann–Whitney *U* test or Kruskal-Wallis test was used for variables that do not satisfy the assumption of homogeneity of variance. The 10 self-designed questions, anxiety, and resilience scores exhibited a non-normal distribution, as indicated by the Shapiro–Wilk test (*p* < 0.05). Additionally, the corresponding Cronbach’s α coefficients were calculated. Spearman’s correlation analysis was used to evaluate the relationships among the self-designed questions, resilience, and anxiety.

Using the bootstrap-mediated effects test, we hypothesized that resilience mediated between self-imposed questions and anxiety. Therefore, the non-parametric percentile bootstrap method was used to test the mediating effect of resilience between self-designed questions and anxiety (*N* = 5,000) (24, 25). The mediating effect analysis aims to determine the presence of a mediating effect and identify the corresponding parameter type. The significance of the parameter refers to the value of the influence effect. A mediating effect is considered statistically significant when the 95% confidence interval of bootstrap does not include 0 (38). A mediated effect path analysis diagram was produced using Amos 23.0 modeling and analysis. All statistical tests were two-tailed, and *p*-values < 0.05 were considered statistically significant.

### Trustworthiness

We took measures to ensure the credibility of this study, as elaborated in our previous research. These measures included (1) the research team comprised individuals with expertise in conducting mixed-methods, qualitative, or quantitative studies; (2) the principal investigator remained in frequent communication with methodological experts to address challenges in the research process continually; (3) participants validated the findings upon analyzing qualitative data to ensure consistency; and (4) the study adhered strictly to operational procedures throughout its execution.

## Results

### Demographic characteristics

The qualitative study had 20 participants with an average age of 34.47 ± 7.47 years, 75% women, and an average work experience of 9.73 ± 8.85 years. The quantitative study had 210 participants with a mean age of 36.62 ± 8.24 years, 75.24% female, and a mean work experience of 13.55 ± 9.55 years. Demographic data are shown in Table 1.

### Qualitative results

The qualitative study results were divided into three parts: pharmacist psychological status under quarantine, factors influencing pharmacist psychological status, and countermeasures against negative psychological states under quarantine.

### Psychological states of pharmacists under quarantine

The psychological state of the HQHPs under quarantine was identified (Table 2). The participants stated that compared to late 2019, HQHPs had gradually adapted to the impact of the epidemic and quarantine, and their mentality was more stable. However, there were still various factors that caused negative emotions, such as anxiety. After 3 years of the epidemic, HQHPs had gained a lot of experience that could help them to alleviate negative emotions. In the quarantined situation, some participants also mentioned that they reflected on life and felt that the value of life had been sublimated.

**Table 2:** The psychological state of HQHPs under quarantine.

Themes	Quotations
Negative emotions	Although we have experienced several outbreaks, this time I felt very sudden, just like when we were not allowed to leave the building at the beginning, we were still quite anxious *(G1P3).*
	After the third day, I felt that I lacked a spiritual support at home *(P1).*
Adaptation	After so many years, as a young person, I am not so scared of this virus now, I just think that it might be a bad cold, and I will just fight it off *(G3P3).*
	When the epidemic first broke out, I felt very panicky and worried that I would be infected, but now that there is a vaccine and medication, I feel adapted and much better *(P6).*
Experience accumulation	In terms of ourselves, this is controllable, because after all, we have experienced it *(G1P1).*
	It’s okay, after all, I’ve been through it so many times now, and I think I’ve had some experience with it, so it does not seem that bad *(G3P1).*
Active coping	I just use that free time and I would feel so happy to be able to quiet my mind and write *(G2P4).*
	The heart is empty, in fact, it has a little impact on the physical and psychological aspects, but on second thought, we still live a regular life, and it is more important to improve our immunity to deal with the epidemic *(P1).*
	We all have to face it with a positive mindset because when you only face him with a positive mindset, your autoimmunity will be strong and the virus will not be able to attack your body easily, right? *(G2P1).*

G, group; P, participant; For example, G1P1 means the first participant from the first group.

### Multidimensional factors affecting the psychological state of HQHPs

Two main themes were identified regarding the factors influencing the HQHPs’ mental health (Table 3). Among them, positive factors include (1) personal factors, such as good physical state and optimism; (2) supportive interpersonal relationships, such as family companion, connection with friends, and colleague support; and (3) harmonious environment, for example, a family environment. The negative factors include: (1) community defense measures deemed unreasonable, (2) insufficient supplies, (3) anti-epidemic policies perceived as inadequate, (4) unscientific management of public opinion, including opaque information and low credibility of self-media, and (5) other unforeseen emergencies.

**Table 3:** Factors affecting the mental health of HQHPs.

(Sub-)themes	Quotations
**1 Positive factors**	
**1.1 Personal factors**	
Good physical state	*I feel that my body is healthy, my immune system is fine, and that’s important (G2P1).*
Optimism	*I feel like I’m always optimistic (P2).*
	*I think I have an acceptable personality and am relatively optimistic (P7).*
	*I am a relatively optimistic person (P3, P6, P8).*
**1.2 Supportive interpersonal relationships**	
The company of family	*Chat with family members* via *telephone/video can help ease my anxiety (P2).*
	*My parents are relatively positive people, they will comfort you that it is impossible to stay at home because of the epidemic now, let us relax and do not have economic pressure (P6).*
	*Because of the pandemic, I could spend more time with children and communicate with them, which could help them and myself relieve negative emotions (P7).*
Connection with friends	*I would talk to my friends, and as a medical professional myself, I felt it was my duty and responsibility to enlighten them or lead them in a positive direction (P8).*
Support from colleagues	*Because I think most of my colleagues are quite optimistic when it comes to this kind of thing, and although it is true that some of them may not be able to adapt for a period of time, we all encourage each other and enlighten each other, and I feel that this overall atmosphere is still manageable (G1P2).*
**1.3 Harmonious environment**	
Family environment	*After so much experience, I feel that the family’s mentality is more peaceful, and the environment of our family is still relatively harmonious during this period (G2P3).*
**2 Negative factors**	
2.1 Unreasonable community defense measures	*The mood is still affected differently by different levels of management in the community. I think the policies in our neighborhood are too strict and affect normal life (G1P1).*
	*The community told me to sign a letter of commitment，that I could go out and then not be allowed to come back. However, I have two small children at home, so if I go to work and am not allowed to come home, the impact will definitely be great, and I think this policy is unreasonable (G2P1).*
2.2 Inadequate supplies	*Inadequate basic supplies (e.g., food, water) during quarantine was a source of anxiety (P3).*
2.3 Unreasonable anti-epidemic policies	*The epidemic lockdown is one-size-fits-all, and the duration of lockdown is unclear, making it impossible for health workers to participate in front-line work (P4).*
2.4 Unscientific management of public opinion	*The current epidemic prevention policy, anyway, is not very transparent, and people do not get enough information about the social side of the community, and they do not get effective feedback, so it creates a sense of fear (G1P2).*
	*This round of epidemic information has not been released in an open, transparent, and timely manner (P1,P3,P4,P8).*
	*Because the official information is not timely, the public opinion on the internet cannot distinguish between true and false (P2,P5).*
	*There are some flaws in this information disclosure, which then leads to more rumors and then a strong sense of panic among people (G2P2).*
2.5 Other emergencies	*Because I work in the hospital pharmacy, relatives in my family often entrust me with the purchase of medicines. Some people have special conditions that prevent them from cutting off their medication. But because of this period of closure, it would not be possible to get their medication in time, and this matter would cause me a little distress (P4).*
	*My child has serious constipation recently, and I cannot take him out to see a doctor, and then my family has a lot of arguments because of his constipation (P5).*

### Strategies for coping with psychological problems under quarantine

The strategies to cope with psychological problems fell into five broad categories (Table 4). Most of the participants highlighted exercise, such as yoga, as a stress-relief method. Interacting with family or friends was also cited as a mood-enhancing activity. The participation in simple household tasks such as cooking, in addition to participating in personal favorite recreational activities such as watching movies or listening to music, were identified as effective coping strategies. Lastly, involving oneself in work-related tasks to maintain a productive state was mentioned as a means to alleviate negative emotions.

**Table 4:** Coping strategies.

Themes	Quotations
Exercise	*Coping strategies for me is exercise, read a book, listen to music, or making food (P1).*
	*Following Tik Tok to do fat loss exercises, or other exercises, is a very important way to vent emotions (G3P4).*
Interpersonal interaction	*The main way to relieve anxiety is through family activities, such as playing games with children (P2).*
	*We can communicate with friends by video, and I also drink with my friends by video, which is quite relaxing (G1P2).*
	*Video chat with other brothers to have a heart to heart talk (G1P4).*
	*There are so many things that we can play with, and even though I’m at home isolation, I have not lost touch with my friends, and that’s one of the things that makes me happy. Modern technological advances have allowed us to play together even when we are not together (G3P2).*
Do the housework	*Do the housework, tidy up the clothes, do the cooking, I think that’s pretty good too (G2P2).*
Entertainment	*For me, listen to music, watch films, and playing with children are the way to ease (P5).*
	*Dancing at home, singing, and watching some funny films with my family, I think it’s great to watch films where everyone laughs and has fun and it’s a way to lighten the mood (G3P2).*
	*When I take a break, I read something I like, then listen to music, occasionally watch a film, it’s all very relaxing (G2P1).*
Maintaining working conditions	*Doing something related to work at home, like reading the literature, can distract me from anxiety and confusion (P4).*

### Quantitative results

**Figure 2:**
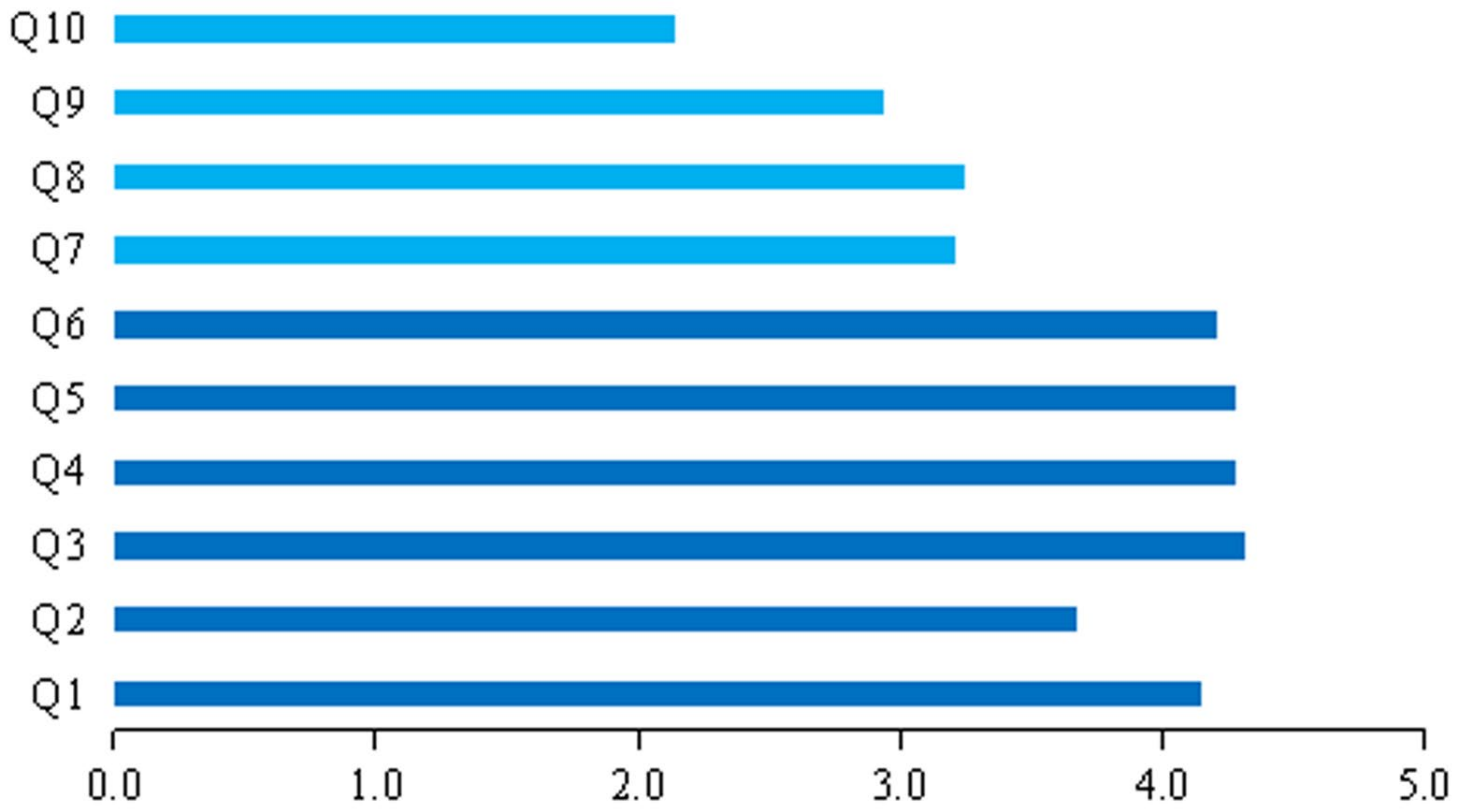
Evaluation of HQHPs on impact factors. Q1: I think my body state enough to deal with in the past few days; Q2: I think I’m naturally optimistic; Q3: I feel very supported by my family; Q4: I think my friends are very supportive; Q5: I get along well with my coworkers; Q6: I think my family environment is very harmonious; Q7: I think the epidemic prevention management in the community is very scientific; Q8: I think the epidemic prevention and control policies are very scientific; Q9: I think the public opinions on epidemic prevention and control are full of positive energy; Q10: I have a stronger religious faith.

In the quantitative part of the study, we implemented a 10-item additional survey based on the qualitative research that defined the role of personal factors, interpersonal relationships, the surrounding environment, community management, epidemic prevention policies, and public opinion management in influencing HQHPs’ mental health (Figure 2). We also used GAD-7 and CD-RISC by a web-based questionnaire to investigate the mental health status of hospital pharmacists. Cronbach’s alpha indicates relatively high internal reliability for the three parts of the questionnaire (Cronbach’s α = 0.839 for the 10 self-designed questions, α = 0.963 for the Chinese version of CD-RISC-25, and α = 0.929 for GAD-7), indicating good reliability of questionnaires in quantitative studies.

We used the Kaiser-Meyer-Olkin (KMO) index to determine if the data were suitable for factor analysis (26). The value of KMO was 0.839, which is above the threshold of 0.7. To facilitate subsequent studies, we conducted an exploratory factor analysis (EFA) on the 10 self-designed questions. EFA revealed two factors with a cumulative explained variance of 65.896%, where Q1-Q6 had higher scores, and this higher level of agreement represents a positive personal state or surrounding environment, thus defining them as positive factors. Whereas, Q7–Q10 had relatively lower scores and lower levels of agreement showed a negative external environment, thus defining them as negative factors (Supplementary Table S4).

### Mental health status of HQHPs

CD-RISC-25 scores for HQHPs ranged from 23 to 100, with a median score of 66 [55.75, 74], which is in the range of low resilience. As shown in Supplementary Table S5, the resilience was significantly higher in the male group than in the female group (*p* = 0.006). There were no significant differences in other demographic variables. In terms of anxiety, the median GAD-7 score was 5 [2, 7], which is on the mild anxiety level. There were no significant differences in anxiety scores between all demographic variables.

### HQHPs’ evaluation of impact factors

The findings indicated that 80% of HQHPs affirmed being in good physical condition and sufficiently resilient at the physiological level to cope with the impacts of epidemic containment. A substantial majority (80% or more) of HQHPs reported having a harmonious family living environment supported by family members, friends, and colleagues. However, only 46.34% expressed strong optimism, 21.14% did not consider themselves naturally optimistic, and 32.52% had reservations. Furthermore, the HQHPs expressed concerns about the perceived lack of preventive and control measures at the scientific level and deemed government-level prevention and control policies unreasonable. The management of public opinion at the social level could have been more transparent, with only 38.57%, 45.70%, and 27.62% of the respondents agreeing with the reasonableness of these three aspects, respectively. In particular, a small percentage of HQHPs (7.53%) reported having strong religious beliefs. Detailed quantitative findings are presented in Figure 2.

### Correlation analysis of self-designed questions, resilience, and anxiety

The Shapiro–Wilk test showed that the data conform to nonnormality. Spearman’s correlation analysis showed that positive factors (Q1–Q6) and negative factors (Q7–Q10) were all positively correlated with CD-RISC-25 scores and negatively correlated with the GAD-7 score. There was a significant negative correlation between resilience and anxiety (Spearman correlation *r* = −0.451, *p* < 0.001; Supplementary Table S6).

### The mediating effect of psychological resilience

Following mediating effects analysis, for positive factors (Q1–Q6), the total and indirect effects on anxiety were significant (*p* < 0.01), with a mediating effect confidence interval of [−1.818, −0.384] (Table 5). The indirect impact accounted for 39.01%, indicating partial mediation by resilience. Similarly, for negative factors (Q7–Q10), total and indirect effects on anxiety were significant (*p* < 0.01), with a mediating effect confidence interval of [−1.174, −0.430] (Table 6). Resilience played a partial mediating role, representing 82.87% of the total effect, while the direct effect of negative factors on anxiety was non-significant (*p* > 0.05).

**Table 5:** The mediating effect of resilience between positive factors and anxiety.

Path	(Effect)	(SE)	LLCI (95%)	ULCI (95%)	t (p)	Effect value (%)
a:PF → RES	15.51	1.286	12.979	18.048	12.07 (<0.001)	
b:RES → ANX	−0.07	0.019	−0.104	−0.029	−3.49 (<0.001)	
c:PF → ANX (total effect)	−2.64	0.363	−3.362	−1.932	−7.30 (<0.001)	
d:PF → RES → ANX (indirect effect)	−1.03	0.367	−1.818	−0.384		39.01
e:PF → ANX (direct effect)	−1.62	0.461	−2.525	−0.708		61.36

PF, Positive Factors; RES, Resilience; ANX, Anxiety; LLCI (95%), Lower level of the 95% confidence interval; ULCI (95%), Upper level of the 95% confidence interval.

**Table 6:** The mediating effect of resilience between negative factors and anxiety.

Path	(Effect)	(SE)	LLCI (95%)	ULCI (95%)	t (*p*)	Effect value (%)
a:NF → RES	7.25	1.042	5.199	9.308	9.41 (<0.001)	
b: RES → ANX	−0.11	0.017	−0.138	−0.072	−5.29 (<0.001)	
c:NF → ANX (total effect)	−0.92	0.273	−1.461	−0.384	−4.95 (<0.001)	
d:NF → RES → ANX (indirect effect)	−0.761	0.192	−1.174	−0.430		82.72
e: NF → ANX (direct effect)	−0.16	0.279	−0.709	−0.709		17.39

NF, Negative Factors; RES, Resilience; ANX, Anxiety; LLCI (95%), Lower level of the 95% confidence interval; ULCI (95%), Upper level of the 95% confidence interval.

**Figure 3:**
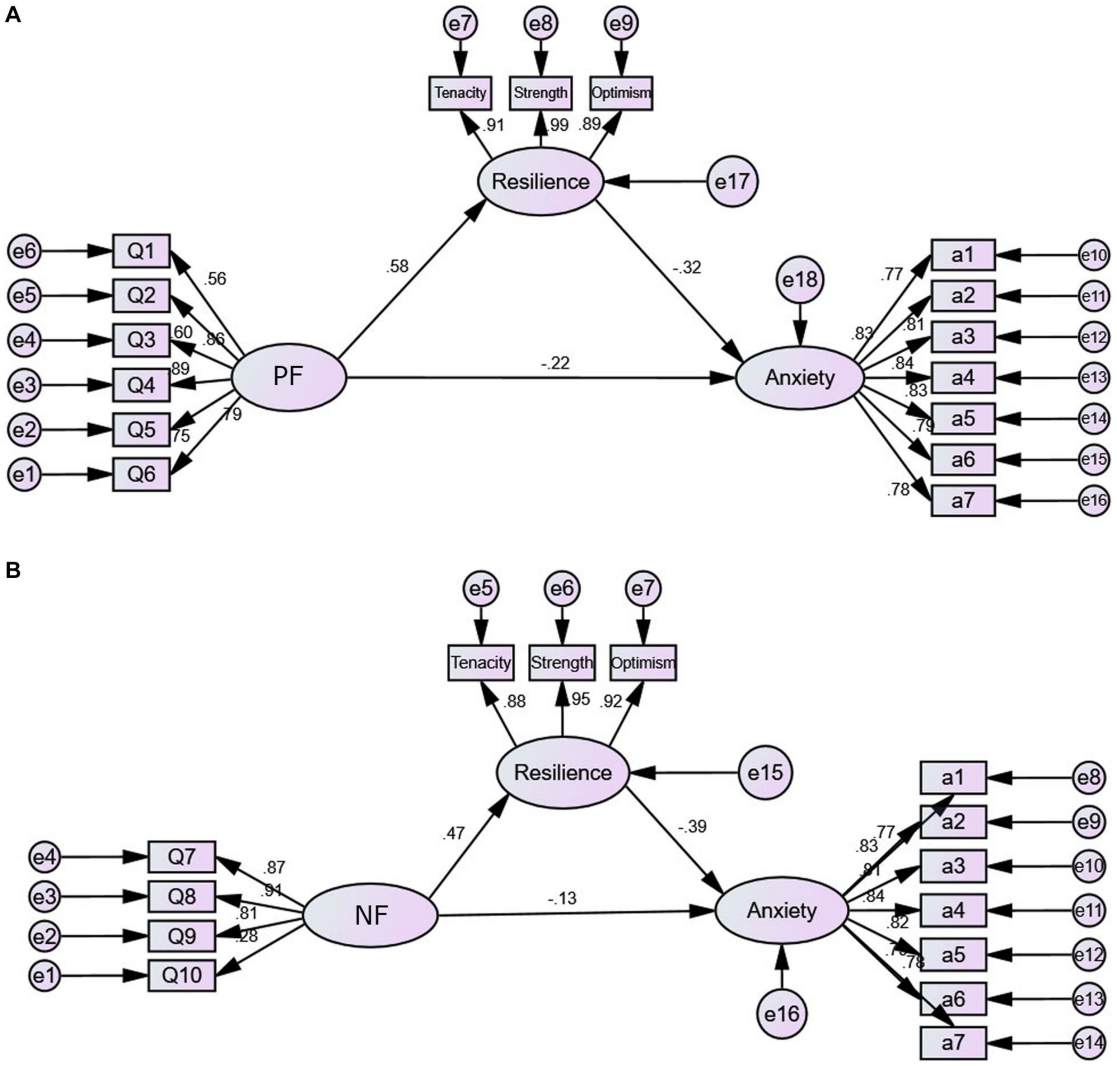
A mediating model of resilience between positive factors **(A)**, negative factors **(B)** and anxiety. PF, Positive factors; NF, Negative factors.

Amos was used to construct a structural equation model, with resilience serving as a mediator variable (Figure 3). Regarding positive factors (Q1–Q6), the results indicated that the path coefficients in the model were statistically significant (*p* < 0.01), and the fit indices for the structural equation model were as follows: CMIN/DF = 3.524, RMSEA = 0.110. Specifically, Q7–Q10 demonstrated a positive impact on resilience (β = 0.58, *p* < 0.01), while resilience showed a negative effect on anxiety (β = −0.32, *p* < 0.01). Concerning negative factors, the path coefficient of the model was also statistically significant (*p* < 0.01), and the fit indices were deemed acceptable: CMIN/DF = 2.111, RMSEA = 0.073. In particular, Q7–Q10 showed a positive influence on resilience (β = 0.47, *p* < 0.01), and resilience had a negative effect on anxiety (β = −0.39, *p* < 0.01).

## Discussion

This study is one of the few mixed-method investigations to examine the mental health challenges faced by HQHPs in the later stages of the COVID-19 pandemic. The unique circumstances encountered by the participants can be attributed to several factors: (1) they remained under home quarantine for up to 2 weeks even after quarantine measures were lifted globally; (2) the study city, Zhengzhou, being a megacity, faced challenges in the supply of essential living materials during the quarantine period (27, 39); (3) unlike prior quarantines where medical personnel, including hospital pharmacists, were allowed to move freely with work permits, this time they were subjected to strict restrictions; and (4) despite the crucial role pharmacists play in epidemic prevention and control, their contributions were occasionally overlooked (13). Consequently, we believe that the findings of this study contribute valuable information to a broader understanding of the mental health of medical staff in the context of pandemics like COVID-19.

The qualitative research findings shed light on the impact of home isolation on the mental well-being of hospital pharmacists and offer an initial exploration of the coping measures they employ. The factors influencing home quarantine on the mental health of HQHPs were identified and categorized into positive and negative factors. The positive factors were optimism, family companionship, and connections with friends. These findings are consistent with existing research, highlighting the role of personal resources such as optimism, prosocial behavior, and resilience in effectively mitigating the adverse consequences of the pandemic on mental health (40–42). In addition, the importance of family companionship and connections with friends in alleviating mental stress has also been affirmed in other studies (43, 44).

Negative factors include insufficient supplies, inadequate information, and other emergent situations. The substantial impact of scarce basic supplies on psychological distress has been well-documented in previous studies (45). In response, relevant government departments must ensure that quarantined households have ample supplies to meet basic needs, delivered promptly, and ideally coordinated in advance with conservation and redistribution plans to prevent resource depletion.

Since the onset of COVID-19, there has been increased demand and attention for pandemic-related information. Information reception generally falls into two categories: official and unofficial sources. Official sources involve government disclosures and expert voices in medical and other fields. Unofficial sources include Internet users’ opinions, gossip, and conversations with family and friends, both online (e.g., WeChat, phone calls) and offline (face-to-face). In this study, numerous participants indicated dissatisfaction with the official release of information, causing a reliance on social media for information, which may contain misinformation and disinformation detrimental to physical and mental health. In line with WHO recommendations, member states should develop action plans to manage the infodemic by promoting the timely dissemination of accurate information based on science and evidence, particularly to high-risk groups, while combating misinformation and respecting freedom of expression (46). HQHPs in quarantine may also be concerned about staff shortages that cause additional work for colleagues. Other emergencies, such as difficulty accessing regular medical care and prescriptions, emerged as challenges for some participants.

In the second stage, GAD-7 and CD-RISC-25 were used to assess the mental health levels (anxiety and resilience) of HQHPs. The results revealed that the HQHPs exhibited mild anxiety levels and resilience scores within the range of low resilience. In particular, at the time of the study, the epidemic had persisted for nearly 3 years. On the one hand, the public’s attitude toward the epidemic had evolved from initial panic and anxiety to adaptation and acceptance, resulting in generally mild anxiety levels among HQHPs (47). However, multiple sporadic cases and local outbreaks, coupled with uncertainty about when the outbreak would end, contributed to a reduction in the psychological resilience of the public (40). At this stage, we explored the correlations between the influencing factors identified in the first stage and the levels of resilience and anxiety. The results confirmed that, except for religious belief, the other factors were positively and negatively correlated with resilience and anxiety, respectively. These findings have significant value in preventing and reducing psychological issues among pharmacists.

This study has several strengths and weaknesses. Strengths include enhancing knowledge of hospital pharmacists’ psychological state post-epidemic, a specific and representative target population and scenario, and using an embedded mixed methods approach for robust results. However, weaknesses include a relatively small quantitative sample size, a need for in-depth exploration of resilience mediating effects, a limited geographic scope to one province in central China, and the potential for subjective biases in data interpretation. Researchers should exercise caution, ensuring transparency in their methodology to mitigate these limitations.

## Conclusion

Our study reveals significant correlations between the psychological resilience and anxiety of HQHPs and their traits, the micro-environment of survival, and the macro-environment. Except for religious beliefs, all first-stage influencing factors are positively correlated with CD-RISC-25 and negatively correlated with GAD-7. This underscores the importance of maintaining positive relationships with family and friends during home sequestration in the face of public health emergencies. Furthermore, our research explores coping strategies used by HQHPs to alleviate anxiety during stay-at-home conditions, such as exercise, yoga, watching films, listening to music, cooking, and playing with children. These findings offer insight into safeguarding the psychological well-being of healthcare workers in future public health emergencies.

## Data availability statement

The raw data supporting the conclusions of this article will be made available by the authors, without undue reservation.

## Ethics statement

Ethics was approved by the First Affiliated Hospital of Zhengzhou University Institutional Review Board (No. 2022-KY-0736). The studies were conducted in accordance with the local legislation and institutional requirements. Written informed consent for participation in this study was provided by the participants' legal guardians/next of kin.

## Author contributions

ZY: Investigation, Methodology, Writing – original draft, Writing – review & editing. XW: Data curation, Investigation, Writing – original draft. XL: Data curation, Investigation, Writing – original draft. HF: Funding acquisition, Methodology, Writing – review & editing.
