# Supplementary material for: Hospital pharmacists’ mental health during home isolation in the post-pandemic era of COVID-19: influencing factors, coping strategies, and the mediating effect of resilience
Source: Front Public Health. 2024 Jan 31;12:1268638. doi: 10.3389/fpubh.2024.1268638 (PMC10864583; doi:10.3389/fpubh.2024.1268638)
Supplement: Supplementary file 1 [file Data_Sheet_1.docx]

**Table S1** Self-designed questionnaire

| **Question items** | **Specific description** | **totally**  **disagree** | **disagree** | **partially agree** | **agree** | **totally agree** |
| --- | --- | --- | --- | --- | --- | --- |
| Q1 | I think my body state enough to deal with in the past few days | 1 | 2 | 3 | 4 | 5 |
| Q2 | I think I'm naturally optimistic | 1 | 2 | 3 | 4 | 5 |
| Q3 | I feel very supported by my family | 1 | 2 | 3 | 4 | 5 |
| Q4 | I think my friends are very supportive | 1 | 2 | 3 | 4 | 5 |
| Q5 | I get along well with my coworkers | 1 | 2 | 3 | 4 | 5 |
| Q6 | I think my family environment is very harmonious | 1 | 2 | 3 | 4 | 5 |
| Q7 | I think the epidemic prevention management in the community is very scientific | 1 | 2 | 3 | 4 | 5 |
| Q8 | I think the epidemic prevention and control policies are very scientific | 1 | 2 | 3 | 4 | 5 |
| Q9 | I think the public opinions on epidemic prevention and control are full of positive energy | 1 | 2 | 3 | 4 | 5 |
| Q10 | I have a stronger religious faith. | 1 | 2 | 3 | 4 | 5 |

**Table S2** Questions used in the interview guide

| **Items** | **Questions** | |
| --- | --- | --- |
| Mental health | Q1.How was your mental health status during your home quarantine? | |
|  | Q2.How has your mental health status changed compared to the beginning of the outbreak (late 2019 or 2020)? | |
| Influencing factors | What factors have influenced your mood? | Q3. Which of your own personal traits (psychology, personality, illness) affect your mental health? |
|  |  | Q4. What are the influences of those around you, parents, other family members, friends, etc.? |
|  |  | Q5. What are the influences of your home environment, work environment, community environment, etc.? |
|  |  | Q6. Are there any influences from some general environmental factors, such as epidemic prevention policies, public opinions related to epidemic prevention, personal religious beliefs, etc.? |
|  |  | Q7. Thinking back on such a journey from the end of 2019, to now, how has your level of mental health changed? |
| Coping Strategies | Q8. Please talk about, during the lockdown period, what useful strategies you take to relieve the psychological burden? | |

**Table S3** Consolidated criteria for reporting qualitative studies (COREQ): 32-item checklist

| No Item | Guide questions/description | Answers |
| --- | --- | --- |
| **Domain 1: Research team and reflexivity** | | |
| Personal Characteristics | | |
| 1. Interviewer/facilitator | Which author/s conducted the interview or focus group? | Zhao Yin, Xiangyu Wang, and Xiaojing Lu |
| 2. Credentials | What were the researcher’s credentials? E.g. PhD, MD | Zhao Yin, MS; Xiangyu Wang, MS; Xiaojing Lu, MS; Hang Fu, PHD |
| 3. Occupation | What was their occupation at the time of the study? | Zhao Yin and Xiaojing Lu are hospital pharmacists; Xiangyu Wang and Hang Fu are hospital administrative staff |
| 4. Gender | Was the researcher male or female? | Zhao Yin, Male; Xiangyu Wang, Male; Xiaojing Lu, Female; Hang Fu, Male |
| 5. Experience and training | What experience or training did the researcher have? | Theoretical training and experience of conducting several qualitative studies with other groups. |
| Relationship with participants | | |
| 6. Relationship established | Was a relationship established prior to study commencement? | Yes |
| 7. Participant knowledge of the  interviewer | What did the participants know about the researcher? e.g. personal goals, reasons for doing the research | Reasons for doing the research |
| 8. Interviewer characteristics | What characteristics were reported about the interviewer/facilitator? e.g. Bias, assumptions, reasons and interests in the research topic | Reasons and interests in the research topic |
| **Domain 2: study design** | | |
| Theoretical framework | | |
| 9. Methodological orientation and Theory | What methodological orientation was stated to underpin the study? e.g. grounded theory, discourse analysis, ethnography, phenomenology, content analysis | Phenomenology. |
| Participant selection | | |
| 10. Sampling | How were participants selected? e.g. purposive, convenience, consecutive, snowball | Purposive |
| 11. Method of approach | How were participants approached? e.g. face-to-face, telephone, mail, email | phone calls or video chat. |
| 12. Sample size | How many participants were in the study? | Quantitative stage: 210  Qualitative stage: 20 |
| 13. Non-participation | How many people refused to participate or dropped out? Reasons? | No one refused and dropped out. |
| Setting | | |
| 14. Setting of data collection | Where was the data collected? e.g. home, clinic, workplace | phone calls or video chat. |
| 15. Presence of non-participants | Was anyone else present besides the participants and researchers? | No |
| 16. Description of sample | What are the important characteristics of the sample? e.g. demographic data, date | Yes, demographic data. |
| Data collection | | |
| 17. Interview guide | Were questions, prompts, guides provided by the authors? Was it pilot tested? | Yes |
| 18. Repeat interviews | Were repeat interviews carried out? If yes, how many? | No |
| 19. Audio/visual | Did the research use audio or visual recording to collect the data? | Audio recording |
| 20. Field notes | Were field notes made during and/or after the interview or focus group? | Yes |
| 21. Duration | What was the duration of the interviews or focus group? | 25-50 min |
| 22. Data saturation | Was data saturation discussed? | Yes |
| 23. Transcripts returned | Were transcripts returned to participants for comment and/or correction? | Yes |
| **Domain 3: analysis and findingsz** | | |
| Data analysis | | |
| 24. Number of data coders | How many data coders coded the data? | Two |
| 25. Description of the coding tree | Did authors provide a description of the coding tree? | Yes |
| 26. Derivation of themes | Were themes identified in advance or derived from the data? | Derived from the data |
| 27. Software | What software, if applicable, was used to manage the data? | NVIVO 12 |
| 28. Participant checking | Did participants provide feedback on the findings? | Yes |
| Reporting | | |
| 29. Quotations presented | Were participant quotations presented to illustrate the themes / findings? Was each quotation identified? e.g. participant number | Yes |
| 30. Data and findings consistent | Was there consistency between the data presented and the findings? | Yes |
| 31. Clarity of major themes | Were major themes clearly presented in the findings? | Yes |
| 32. Clarity of minor themes | Is there a description of diverse cases or discussion of minor themes? | Yes |

**Table S4** Results of exploratory factor analyses for for 10 self-designed questions

| **Question items** | **Factor 1** | **Factor 2** |
| --- | --- | --- |
| Q1 | **0.580** | 0.333 |
| Q2 | **0.586** | 0.489 |
| Q3 | **0.872** | 0.038 |
| Q4 | **0.876** | 0.113 |
| Q5 | **0.846** | 0.110 |
| Q6 | **0.809** | 0.152 |
| Q7 | 0.339 | **0.823** |
| Q8 | 0.256 | **0.853** |
| Q9 | 0.230 | **0.833** |
| Q10 | -0.115 | **0.517** |
| Variance explained (%) | 38.247 | 27.650 |
| Cumulative variance (%) | 38.247 | 65.897 |

**Table S5** CD-RISC-25 and GAD-7 scores for the HQHPs in quantitative stage

| **Item** | **Classification** | CD-RISC-25 score^a^ | ***P value*** | GAD-7 score^a^ | ***P value*** |
| --- | --- | --- | --- | --- | --- |
| **Gender** | Male | 72.5[58.3; 85.8] | 0.007** | 4[2; 7] | 0.302 |
|  | Female | 64 [54.0; 73.0] |  | 5[2; 7] |  |
| **Age** | ≤35 | 66 [54.0; 74.0] | 0.875 | 4[2; 7] | 0.990 |
|  | 36-50 | 66 [55.0; 75.0] |  | 6[1; 7] |  |
|  | >50 | 65 [57.5; 77.0] |  | 5[2; 7] |  |
| **Educational level** | Technical secondary school | 69 [59.0; 73.5] | 0.505 | 4[3; 7] | 0.972 |
|  | Junior college | 57 [48.0; 75.0] |  | 5[2; 7] |  |
|  | Undergraduate | 64 [55.8; 74.0] |  | 6[2; 7] |  |
|  | Postgraduate | 67 [59.0; 74.8] |  | 4[2.3; 7] |  |
| **Working years** | <10 | 67 [55.0; 74.0] | 0.985 | 4[2; 7] | 0.330 |
|  | ≥10 | 65 [56.0; 75.0] |  | 6[2; 7] |  |
| **Marriage status** | Unmarried | 64 [52.0; 74.5] | 0.688 | 4[1.3; 7] | 0.309 |
|  | Married | 66 [56.0; 74.0] |  | 6[2; 7] |  |
|  | Divorced | 70 [60.0; 80.0] |  | 5[0; 7] |  |
| **Professional title** | Junior professional post | 68 [53.5; 79.3] | 0.376 | 4[2; 7] | 0.841 |
|  | Intermediate professional post | 64 [54.0; 73.0] |  | 5[2; 7] |  |
|  | Associate senior professional post | 66 [60.0; 74.0] |  | 4[1; 7] |  |
|  | Senior professional post | 71.5 [63.5; 89.0] |  | 5[0.75; 7] |  |
| **Days of continuous quarantine** | 0–7 days | 64 [54.0; 76.5] | 0.397 | 4[2; 7] | 0.533 |
|  | 8–15 days | 67 [57.0; 74.0] |  | 5[2; 7] |  |

a:the results are reported as median with [25% lower quartile; 75% upper quartile]; ***P* < 0.01.

**Table S6** Correlation analysis of self-designed questions, resilience, and anxiety

|  | X1 | X2 | Resilience | Anxiety |
| --- | --- | --- | --- | --- |
| X1 | 1.000 |  |  |  |
| X2 | .425^**^ | 1.000 |  |  |
| Resilience | .641^**^ | .396^**^ | 1.000 |  |
| Anxiety | -.477^**^ | -.207^**^ | -.451^**^ | 1.000 |

**Correlation is significant at the 0.01 level (2-tailed).


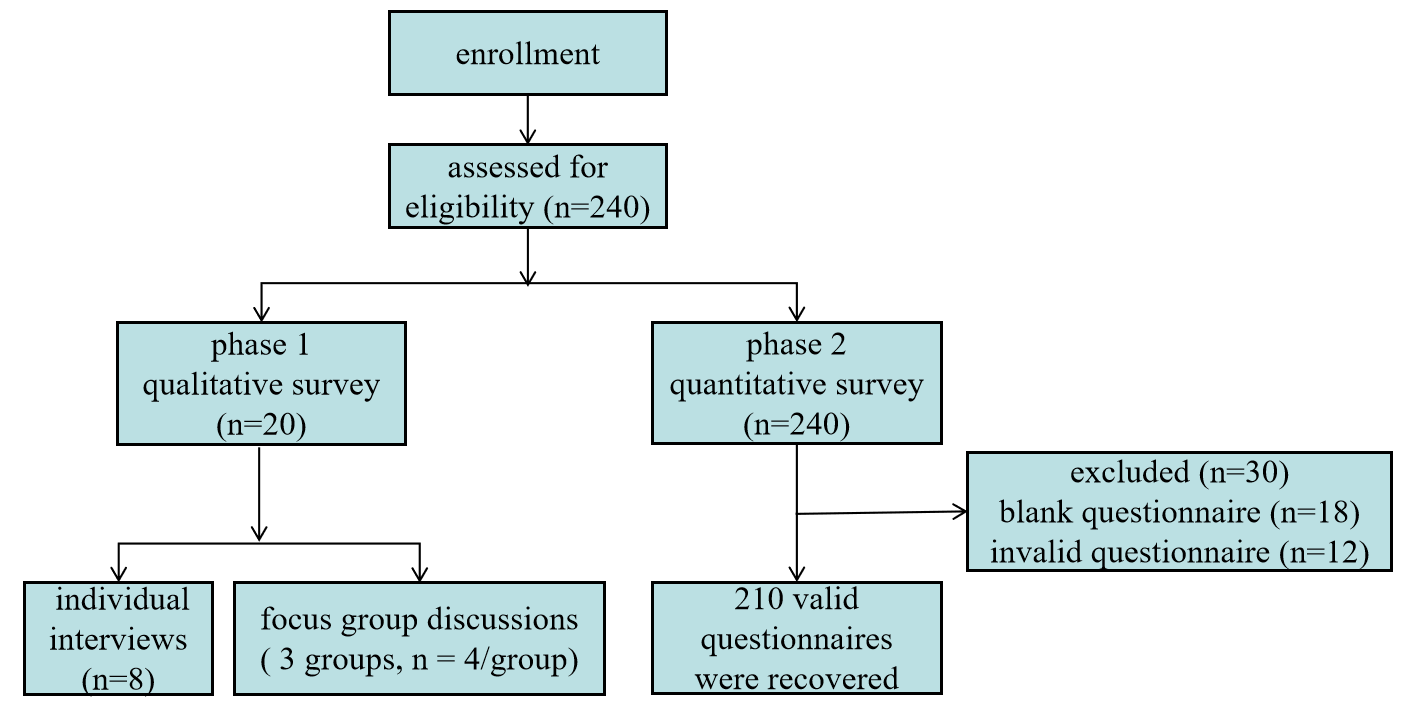


Figure S1 CONSORT diagram reflecting flow of study participants through the study.
